# Deep Venous Thrombosis of Non-operative Clavicle Fracture Following Low-Energy Injury Mechanism With Extension Into the Radial Vein: A Case Report

**DOI:** 10.7759/cureus.37894

**Published:** 2023-04-20

**Authors:** Jennifer Blum, Jacqueline Krumrey

**Affiliations:** 1 Orthopedic Surgery, Western University of Health Sciences, College of Osteopathic Medicine of the Pacific - Northwest, Lebanon, USA; 2 Orthopedics and Traumatology, Good Samaritan Regional Medical Center, Corvallis, USA

**Keywords:** radial vein thrombosis, non-operative management, clavicle fracture, mid shaft clavicle fracture, deep vein thrombosis (dvt), venous thromboembolism (vte)

## Abstract

Clavicle fractures are relatively common with the majority treated non-operatively. However, venous thromboembolism (VTE) in association with these fractures is rare, despite conservative treatment involving immobilization rather than surgical intervention. Surgery is a risk factor for thromboembolism and therefore more common when clavicle fractures are treated operatively. There have been a few published case reports of VTE following clavicle fractures that were managed non-operatively. Here we present a unique case of VTE involving the subclavian, brachial, and radial vein following a low-energy injury, with radial involvement being the most distal to date. A literature review is also presented to compare locations of VTE, injury factors, and timeline from injury to VTE presentation.

## Introduction

Thromboses associated with clavicle fractures are rare, and most cases are due to penetrating trauma or high-energy mechanism [1-4]. Upper extremity venous thromboembolisms (VTEs) only comprise 1-4% of all VTEs [5]. Additionally, there is limited to no evidence suggesting any association between VTE and immobilization of the upper extremity, as when clavicle fractures are treated with a sling [5]. There are thus no official recommendations for VTE prophylaxis in regard to upper extremity immobilization [5]. VTE prophylaxis is only indicated for upper extremity fractures with risk factors for VTE such as hypercoagulability or bed confinement [5].

There are, however, a few cases reported of VTE sustained after non-operative treatment of low-energy clavicle fractures [1,6-8]. Of these cases, the subclavian, axillary, and brachial veins were most commonly involved [1,6-8]. This study aimed to provide another rare example of VTE after a low-energy, non-operative clavicle fracture.

## Case presentation

A 79-year-old female presented to the emergency department after she tripped at home and fell down four steps, sustaining a right clavicle fracture. She was provided a sling and remained non-weight bearing without any shoulder range of motion exercises. She then presented to the clinic 14 days later. Medical history is significant for a right rotator cuff repair 16 years prior, hypertension, hyperlipidemia, and osteoporosis. She has a history of smoking but quit 50 years ago.

**Figure 1:**
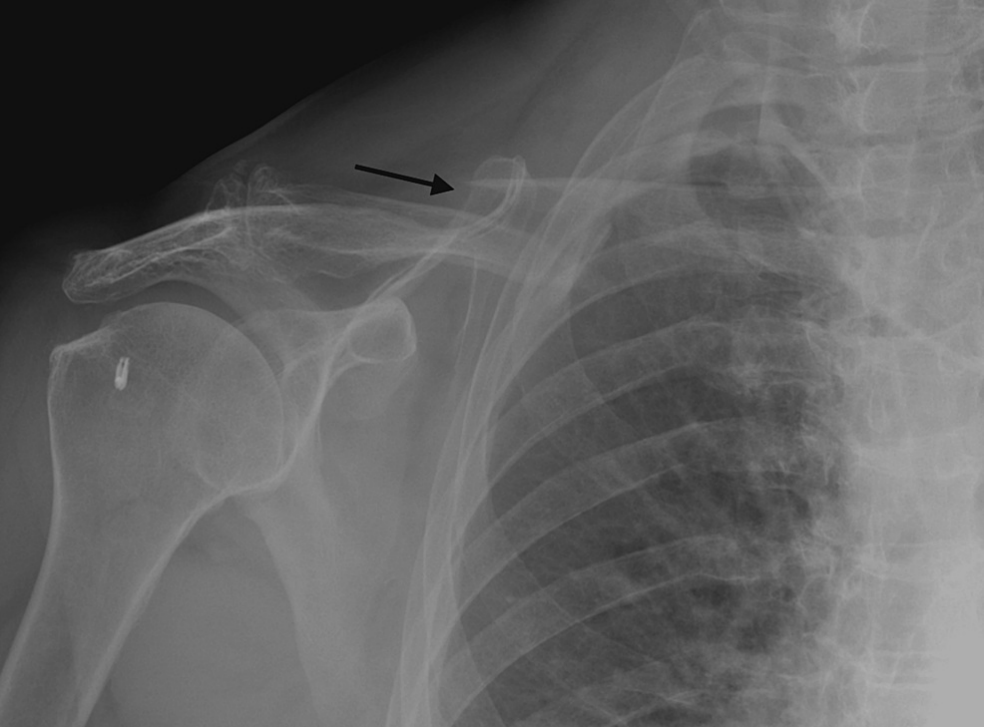
Initial radiograph of clavicle injury. AP radiograph of the right clavicle demonstrating oblique middle third fracture.

On physical examination, she was acutely tender to palpation over the right shoulder and trapezius with mild swelling over the middle third of the right clavicle without ecchymosis. She was neurovascularly intact without paresthesias, had a brisk radial pulse, and had intact motor to anterior interosseous, posterior interosseous, and ulnar nerves. Initial x-ray revealed a midshaft oblique right clavicle fracture with superior displacement and a small butterfly fragment (Figure 1). No shortening was present. She was treated non-operatively and instructed to wean herself out of the sling and begin a gentle range of motion at the elbow four times daily followed by shoulder range of motion exercises over the next week. Follow-up was planned for four weeks.

Twenty days after being seen in the clinic, she presented to her primary care provider (PCP) with new onset of right upper extremity swelling and increased pain. The edema was found to extend from the shoulder to the medial elbow. She was otherwise neurovascularly intact. A venous ultrasound (US) was ordered, revealing a non-occlusive venous thrombosis of the distal subclavian vein, proximal brachial vein, and a small segment of the radial vein in the proximal forearm. She was started on rivaroxaban for anticoagulation.

**Figure 2:**
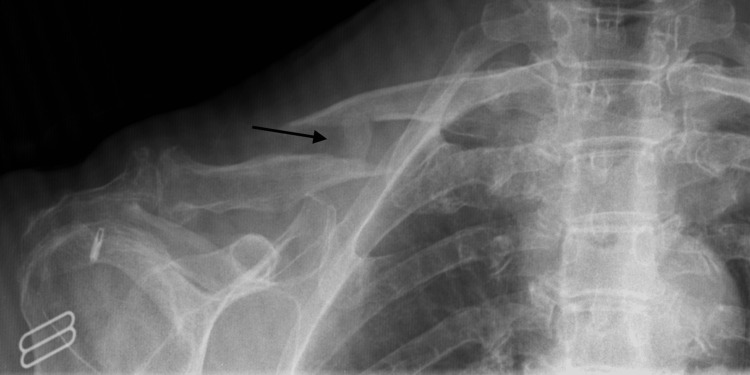
Six-week follow-up clavicle radiograph. Radiograph of right clavicle at six-week follow-up appointment demonstrating early callus formation.

At her first follow-up visit, six weeks after the initial injury and five days after being diagnosed with VTE, she began to use her right arm for activities and swelling had improved. The only remarkable finding on physical examination was decreased active range of motion of the shoulder and trapezial trigger points. Repeat films revealed early callus formation over the dorsal and lateral aspects of the fracture site (Figure 2). Physical therapy consisting of range of motion exercises was recommended to reduce her shoulder stiffness. Once normal range of motion was achieved, she was advised to start strengthening exercises.

She was seen again in the clinic three months after her injury, where she was found to be non-tender over the clavicle. At this same time repeat venous US still showed evidence of non-occlusive thrombosis in the subclavian and brachial vein, with resolution of the thrombosis in the radial vein. Given these results, she was prescribed another three months of anti-coagulation followed by repeat US. Venous US obtained seven months after the initial injury revealed resolution of previous thromboses and anti-coagulation was discontinued.

## Discussion

Thromboses can either be an acute complication following clavicle fracture due to transection of the veins by the displaced fragments or a subacute complication caused by compression of the veins by hypertrophic callus development [9]. The current literature consists of subacute VTE presentations, ranging from one week to two months post-injury, with one week being the most common [1-4,6-9]. All reported thromboses occurred in middle third clavicle fractures, which is consistent with this case presentation. This is likely due to the anatomic relationship between the fracture site and nearby vascular structures, where the subclavian vessels lie just inferior to the middle third of the clavicle.

We found four cases of VTE following clavicle fractures that occurred following moderate or severe trauma which were treated conservatively. These included fractures sustained during marital arts practice [1], unspecified type of sports [2], skiing [3], and a high-speed motorcycle crash [4]. The incidence of VTE following these mechanisms is likely related to the severity of endothelial injury sustained as a part of Virchow’s triad, leading to increased risk of thromboses relative to mild trauma, such as a ground-level fall. Therefore, our case report likely doesn't involve extensive damage to the endothelium or transection of the veins as the mechanism of VTE formation given the low-energy nature of the inciting trauma.

Three cases involving a low-energy trauma mechanism were found in the literature, in the first case VTE was diagnosed two months and 15 days following injury in an atrophic non-union [9]. This was the longest reported delay from injury to VTE. In the second case, non-union associated with VTE after low-energy mechanism was reported in the context of Paget-Schroetter syndrome, where roughly 20 years after initial fracture, VTE was diagnosed [8]. It was found that the non-union led to thoracic outlet syndrome and corresponding subclavian vein compression progressed to thrombosis [8]. In the third case of low-energy trauma, VTE occurred three weeks after a low speed-bicycle accident [6]. This is a similar timeline to this case report, in which VTE occurred 20 days post-injury. Of these three cases the ages of the patients ranged from 48 to 70 years, and this case report being older at 79 years has a higher baseline risk of VTE given the risk increases with increasing age [10,11]. Therefore, age may have played a role in the development of VTE in this case, as patients greater than 40 years of age have a significantly higher risk [11].

Of all reported cases, subclavian and axillary VTE were the most common [1,2,6-9]. Of these, two had additional brachial vein thrombosis and therefore extended distal to the fracture site [2,6]. One case had VTE in the axillary and brachial veins without involvement of the subclavian vein, however, the injury mechanism was high-energy via a fall while skiing [3]. The patient initially presented with diffuse swelling in the extremity, paresthesias, and diminished radial pulse nine days post-injury, making this the most acute presentation of VTE following clavicle fracture found in the literature [3]. It is reasonable to conclude that the VTE in this scenario may be related to the injury itself causing extensive endothelial damage via over-traction of the vein wall rather than the fracture causing transection of the veins given their location as the subclavian vein wasn't involved. There were no cases in our literature review of VTE more distal than the brachial vein, thus our case with extension into the radial vein is a unique presentation.

## Conclusions

Venous thrombosis in association with non-operative clavicle fractures is a rare complication with limited cases reported in literature. It is even less common to develop VTE following a low-energy mechanism given the lack of significant endothelial damage. Perhaps in our case presentation the VTE development was related to the age of the patient as risk for thromboses increases with increasing age in combination with immobilization within a sling. Current guidelines only recommend prophylactic anti-coagulation in cases with inherently increased risk for VTE such as those undergoing surgery, those with malignancy or hypercoagulability. However, here we presented a few cases of thromboses without any obvious risk factors, indicating a high index of suspicion should be maintained for all clavicle fractures regardless of the mechanism of injury. More research is needed to reevaluate if prophylactic anti-coagulation may be beneficial to patients with non-operative clavicle fractures or other upper extremity injuries requiring immobilization, especially in older patients.
